# Low Risk of SARS-CoV-2 Reinfection for Fully or Boosted mRNA Vaccinated Subjects in Sicily: A Population-Based Study Using Real-World Data

**DOI:** 10.3390/vaccines11121757

**Published:** 2023-11-26

**Authors:** Laura Maniscalco, Dario Genovese, Barbara Ravazzolo, Giuseppe Vella, Benedetta Sparacia, Francesco Vitale, Domenica Matranga, Emanuele Amodio

**Affiliations:** 1 Department of Health Promotion, Mother and Child Care, Internal Medicine and Medical Specialties “G. D’Alessandro” (PROMISE), University of Palermo, Via del Vespro 133, 90127 Palermo, Italy; laura.maniscalco04@unipa.it (L.M.); giuseppe.vella02@unipa.it (G.V.); benedetta.sparacia@unipa.it (B.S.); francesco.vitale@unipa.it (F.V.); domenica.matranga@unipa.it (D.M.); emanuele.amodio@unipa.it (E.A.); 2 Unità Operativa Complessa di Epidemiologia Clinica con Registro Tumori, Azienda Ospedaliera Universitaria Policlinico “Paolo Giaccone”, 90127 Palermo, Italy; barbara.ravazzolo@policlinico.pa.it

**Keywords:** SARS-CoV-2, reinfection, COVID-19, population-based analysis, epidemiology, Sicily

## Abstract

**Background**: Reinfections occur as a response to natural infections wanes and novel strains of SARS-CoV-2 emerge. The present research explored the correlation between sex, age, COVID-19 vaccination, prior infection hospitalization, and SARS-CoV-2 reinfection in Sicily, Italy. **Materials and Methods**: A population-based retrospective cohort study was articulated using the vaccination flux from a regional registry and the Sicilian COVID-19 monitoring system of the Italian Institute of Health. Only adult Sicilians were included in the study, and hazard ratios were calculated using Cox regression. **Results**: Partial vaccination provided some protection (adj-HR: 0.92), when compared to unvaccinated individuals; furthermore, reinfection risk was reduced by full vaccination (adj-HR: 0.43), and the booster dose (adj-HR: 0.41). Males had a lower risk than females of reinfection with SARS-CoV-2 (adj-HR: 0.75). Reinfection with SARS-CoV-2 was diminished by hospitalization during the first infection (adj-HR: 0.78). Reinfection risk was higher among those aged 30–39 and 40–49 compared to those aged 18–29, whereas those aged 60–69, 70–79, and 80+ were statistically protected. Reinfection was significantly more frequent during the wild-type–Alpha, Delta, Delta–Omicron, and Omicron dominance/codominance waves compared to the wild type. **Conclusions**: This study establishes a solid base for comprehending the reinfection phenomenon in Sicily by pinpointing the most urgent policy hurdles and identifying some of the major factors. COVID-19 vaccination, one of the most effective public health tools, protects against reinfection, mostly caused by the Omicron strain. Elderly and hospitalized people’s lower risk suggests stricter PPE use.

## 1. Introduction

Coronavirus disease 2019 (COVID-19) is caused by the severe acute respiratory syndrome virus 2 (SARS-CoV-2), which has a lower mortality rate than MERS-CoV and SARS-CoV-1, but also a relevantly higher transmission rate [1]. After its first identification in Wuhan, China, in December 2019 [2], it quickly spread around the World.

As of 22 March 2023, the COVID-19 outbreak has resulted in 760,360,956 confirmed cases, including 6,873,477 deaths [3]. The aforementioned data intensify the need to estimate who is more susceptible to SARS-CoV-2 infection and poses the inevitable question about the persistence of the immunity status.

Through the inclusion of only randomized controlled trials, Graña et al. [4] attempted to provide a comprehensive overview of the evidence concerning the safety and efficacy of COVID-19 vaccines. The primary findings pertain to the effectiveness of vaccines in preventing the progression of severe COVID-19 disease and the development of COVID-19 symptoms. The main conclusion is that numerous COVID-19 vaccines, including those developed using mRNA technology, provide appropriate levels of immune protection against the aforementioned two outcomes [4]. Similarly, the already-mentioned review emphasized the necessity of further research examining the efficacy of the COVID-19 vaccines beyond six months from the date of administration of the first vaccine dose, in light of the postulated decline in vaccine-induced immunity [4].

To date, there is evidence that vaccine-induced immunity to SARS-CoV-2 progressively wanes with time [5,6,7,8,9,10]; at the same time, some studies have shown that those who received COVID-19 vaccines are still better protected than unvaccinated people, with vaccine effectiveness higher than 80% against COVID-19-related hospitalization [8,9,10].

It has been hypothesized that protection against COVID-19 may be reduced by the virus’ constant and rapid mutation, resulting in the emergence of different viral variants. In fact, SARS-CoV-2 is prone to genetic evolution to adapt to human hosts, and these adaptive changes in the genome can, in turn, modify the pathogenic potential of the virus, having an impact on the disease’s severity and transmissibility rate [11]. Furthermore, there is evidence of a decreased immune response towards SARS-CoV-2 variants that are temporally distant from SARS-CoV-2 wild type [12].

The drop in immunity against SARS-CoV-2 and the continuous succession of different strains have contributed to the occurrence of reinfections; this was observed in some research studies on animals and was also documented in humans [13,14]. The first confirmed case of SARS-CoV-2 reinfection was reported in Hong Kong in 2020, 142 days after the first infection [15], followed by many other reports from different countries, such as Ecuador [16,17], Italy [18], India [19], and the United States [20].

There is an ongoing debate on the definition of SARS-CoV-2 reinfection, considering the difficulty of distinguishing between reinfection, relapse, and PCR re-positivity. Yahav et al. [21] recommended referring to reinfection only if the second PCR positivity occurred more than 90 days after the first documented SARS-CoV-2 infection. However, confirmed cases of reinfection have been detected after 19 days [22], suggesting that a strict definition of reinfection might underestimate its incidence.

Numerous population reports of potential SARS-CoV-2 reinfection utilized different inclusion criteria [23,24,25,26]. Population-based public health surveillance data might be employed to carefully examine the above-mentioned cases and guide an evidence-based response. To harmonize evidence, the Centers for Disease Control and Prevention (CDC) recommended a standardized investigative method to identify cases with a high index of suspicion for reinfection [27].

The primary objective of this study was to evaluate the frequency of SARS-CoV-2 reinfections among the residents of Sicily, Italy, differentiating between vaccinated and unvaccinated subgroups; the secondary objective of this study was to assess for factors that may play a role in the recurrence of a second infection with SARS-CoV-2.

## 2. Materials and Methods

### 2.1. Eligibility Criteria

In this observational retrospective cohort study, the Sicilian population was followed-up from 24 February 2020 to 9 September 2022. Sicily is the largest region in Italy and the most populous island in the Mediterranean Sea, with a population of 4,833,705 inhabitants. All participants meeting the subsequent criteria were incorporated into the study: residency in Sicily, having contracted SARS-CoV-2 at least once, availability of swab test date(s), aged 18 years or older, and either unvaccinated or exclusively vaccinated with mRNA vaccine doses.

### 2.2. Methods

The study incorporated data from two distinct population-based surveillance registries, both of which were supplied by the Sicilian Regional Health Office on behalf of the Italian Ministry of Health. In particular, the initial registry comprised data pertaining to Sicilian inhabitants who had received a minimum of one dose of vaccine. This data comprised demographically relevant details, the dates on which each dose was injected, and the exact type of vaccine administered. The second database contained details related to individuals who tested positive for SARS-CoV-2. These data comprised the date of each infection, demographic, and clinical information, as well as the eventual occurrence of hospitalization and death. Data pertaining to each participant were attributed with the Italian identification code, which was converted automatically into an anonymous code to safeguard their privacy. In order to avoid possible reversal of the procedure, the conversion table was deleted.

A reinfection was identified by the presence of two positive RT-PCR samples taken at least 90 days apart with one negative RT-PCR test collected between the first and second episodes, according to the CDC guidelines [27]. The resulting dataset was employed to identify all the individuals conforming to the adopted definition of reinfection; therefore, subjects with two or more positive SARS-CoV-2 PCR tests conducted within an interval of less than 90 days were excluded. The European Centre for Disease Prevention and Control (ECDC) provides free weekly and European-wide access to aggregated data on the percentage of distribution of the most relevant SARS-CoV-2 variants [28]. This information was considered to attribute to each occurrence of infection and reinfection the dominant variant or the two co-dominant variants. Each variant is assumed dominant if, at weekly detection, its prevalence is 80% or more of the total SARS-CoV-2 infections for which the next generation sequencing (NGS) process has been performed; otherwise, if two variants together have a prevalence of at least 80% of the total SARS-CoV-2 infections, that week was considered co-dominated by the two abovementioned variants [29].

### 2.3. Statistical Analysis

Discrete and continuous variables were summarized using means and standard deviations, while categorical variables were represented by counts and percentages. In order to evaluate associations between categorical and quantitative variables, the chi-squared test and *t*-test were applied, respectively.

A descriptive analysis was conducted to draw comparisons between secondary and primary infections. In order to determine incidence and account for delayed occurrence of reinfection, the days at risk encompassed the time span between the first positive SARS-CoV-2 PCR result (which started on 24 February 2020) and either the second positive test or the censoring date, which corresponds to the date of data extraction (9 September 2022).

The determination of person-time at risk for reinfection was as follows: the date of diagnosis of the first episode of infection, as ascertained by a confirmatory test, constituted the entry time at risk for the entire sample. The follow-up period for subjects who were reinfected within 90 days of their first diagnostic test was defined as the timeframe until the date of diagnosis of the second episode. The remaining participants were subject to censorship on 9 September 2022, the date of data extraction. The resulting value, expressed as 100 person-years, was the incidence density of reinfection (95% CI), which was calculated by dividing the number of reinfected subjects by the total person-days at risk. Furthermore, the risk of reinfections was evaluated using a multivariable Cox proportional hazard model. Each hazard ratio (HR) and 95% CI is adjusted for sex, age group, vaccination status, hospitalization during the first infection, and SARS-CoV-2 variant.

Statistical analysis was performed using R for Statistical Computing software (version 4.2.2, Vienna, Austria), and an alpha value lower than or equal to 0.05 was considered statistically significant.

## 3. Results

**Figure 1:**
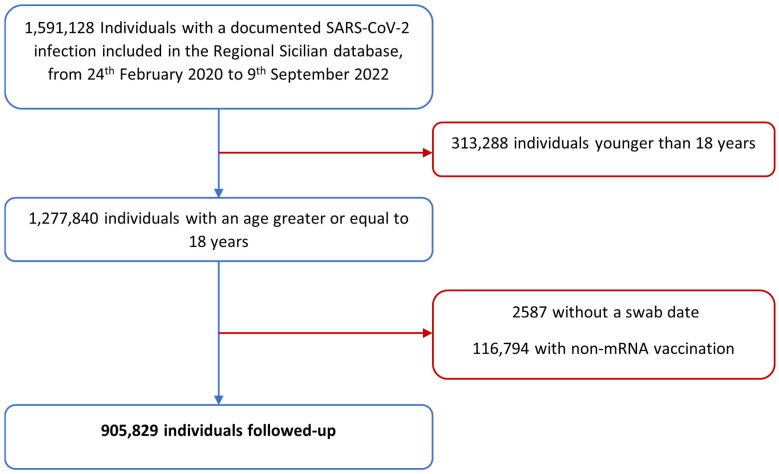
Flowchart representing the Sicilian cohort’s selection process.

The initial cohort included a total of 1,591,128 individuals with a documented SARS-CoV-2 infection registered in the Regional Sicilian database, from 24 February 2020 to 9 September 2022. Thereafter, the following individuals were excluded: 313,288 with an age of less than 18 years, 2587 without a swab date, 116,794 with non-mRNA vaccination, and 252,630 with a reinfection that occurred within 90 days since the previous infection. Hence, a total of 905,829 COVID-19 cases were included in our study (Figure 1).

The incidence of reinfection was 4.3% (*n* = 39,301) during the entire study period. The highest percentage of the first SARS-CoV-2 infection was observed in January 2022 (21.4%), whereas the highest percentage of reinfection was documented in July 2022 (32.6%) (Table 1).

Most of the study population consisted of women (55.2%) and people aged 40 to 49 (19.5%). Only 2.9% of the subjects were hospitalized. Regarding the vaccination cycle, 18.7% were not vaccinated at all, 8.5% received only one dose, 39.1% received the primary full vaccination cycle, and 33.6% received the booster dose. Females were at higher risk of reinfections than males (4.7% vs. 3.9%). Furthermore, people aged ≤59 years old showed a higher risk of reinfection (≥4.3%) compared to older people, as well as hospitalized (5.5%) compared to non-hospitalized patients (4.3%).

Furthermore, those who were fully vaccinated (3.8%) and boosted (1.6%) showed the lowest risk of reinfection (Table 2).

**Figure 2:**
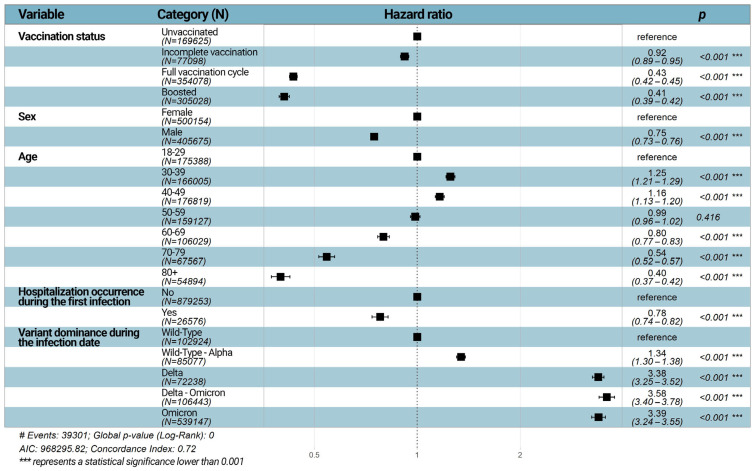
Forest plot of Hazard ratios (HR) according to the multivariable Cox regression analysis. HRs and 95% CIs are estimated for each of the categories related to the variable specified in the homonymous column. The first category for each variable is used to establish the reference category, which has per definition an HR = 1.00 and a 95% CI = 1.00–1.00. In the multivariable model, HRs and 95% CIs are adjusted for all other variables that are taken into account.

As reported in Figure 2, the Cox proportional hazard analysis showed that the risk of reinfection dropped with the increase in the number of vaccine doses. Indeed, one dose of vaccine provided a certain degree of protection against reinfection with SARS-CoV-2 compared to unvaccinated people (adj-HR = 0.92, 95% CI = 0.89–0.95). Furthermore, the hazard ratios of subjects with a full vaccination cycle (adj-HR = 0.43, 95% CI = 0.42–0.44) or with a booster vaccine (adj-HR = 0.41, 95% CI = 0.39–0.42) showed an even lower risk of reinfection compared to unvaccinated subjects. Males had a lower risk of SARS-CoV-2 reinfection than females (adj-HR = 0.75, 95% CI = 0.73–0.76). Individuals aged 30–39 or 40–49 were at higher risk of reinfections compared to subjects aged 18–29 years old (HR = 1.25, 95% CI = 1.21–1.29, HR = 1.16, 95% CI = 1.13–1.20, respectively). In contrast, subjects aged 60–69, 70–79, and 80+ were significantly protected from reinfections compared to people aged 18–29 years old (*p* < 0.001). Moreover, the severity of the first infection seemed to reduce the risk of SARS-CoV-2 reinfection (HR = 0.78, 95% CI = 0.74–0.82). Regarding the variants, compared to the wild type, there was a significantly higher risk of reinfection during wild-type–Alpha (HR = 1.35, 95% CI = 1.31–1.39), Delta (HR = 3.42, 95% CI = 3.29–3.56), Delta–Omicron (HR = 3.61, 95% CI = 3.43–3.80), and Omicron (HR = 3.42, 95% CI = 3.27–3.58) dominance/co-dominance waves (Figure 2).

## 4. Discussion

In the challenging scenario of the SARS-CoV-2 pandemic, the purpose of this study was to determine the frequency of reinfection episodes among the vaccinated and unvaccinated Sicilian inhabitants, as well as to identify any characteristics that may have determined an increase or decrease in the risk of reinfection. The main finding of the study was the lowest risk of reinfection for fully and boosted mRNA-vaccinated people. Furthermore, it was also observed that males, people aged more than 30 years old, people with a more severe first infection, and people with wild-type variant infections were at lower risk of reinfections from SARS-CoV-2. This retrospective cohort study allowed us to investigate a diverse population across various age groups residing within the same region. This approach provided an extensive insight into the phenomenon of SARS-CoV-2 reinfections, enabling a robust analysis of the factors and patterns associated with such occurrences. The length of the study was also noteworthy, with a maximum observation time of 928 days. In addition to providing robust confirmations of prior studies on SARS-CoV-2 reinfections, the study results offer significant additional information.

Vaccination is still being confirmed as a public health strategy for reducing the risk of reinfection in a community. In line with prior research, it is possible to recognize the rising protection from the reinfection event as a function of the number of vaccination doses, with a gradually lower risk rate than in unvaccinated individuals, as shown by the results of this study. Hence, hybrid immunization exhibits greater efficacy than natural immunity itself. Cavanaugh et al. [30] observed that, in the Kentucky resident population, the risk of reinfection was 2.34 times higher among individuals who did not receive any vaccine dose (OR: 2.34, 95% CI: 1.58–3.47), without any statistically significant difference between fully and partially vaccinated patients (OR: 1.56, 95% CI: 0.81–3.0). More recently, Hammerman et al. [31] found that reinfection occurred in 354 of the 83,356 vaccinated patients and in 2168 of the 65,678 unvaccinated subjects (2.46 cases versus 10.21 cases per 100,000 persons per day). Along with prior population-based studies, but with greater robustness and stratification, our data confirm the need to support COVID-19 vaccination programs given the lower risk of second infection among those who received three or more doses [32].

The female population was shown to be more susceptible to reinfection than the male counterpart, with a higher risk of reinfection. Bechmann et al. [33] mention the shift in the M:F ratio between infection and reinfection rates. In fact, whereas primary infections are more prevalent among men, reinfections are more frequent among women. According to the meta-analysis conducted by Flacco et al. [23], the reinfection rate among women is 0.79%, whereas the same rate among men is 0.55%. When analyzing the Ligurian population (North-Western Italy), Piazza et al. [34] found that females have a 17% higher risk of reinfection than males (OR: 1.17, 95% CI: 1.13–1.21, *p* < 0.0001); similarly, in the Abruzzo region (Central Italy), it was found that women have a 32% higher risk than men of contracting a second infection (adj-HR: 1.32, 95% CI: 1.14–1.53). The causes of this shift are poorly understood, but hypotheses can be attempted. Although unexpected, it has been postulated that the increased likelihood of reinfection in women may be attributable to behavioral risk factors, different perceptions of COVID-19 disease, or different working conditions. In fact, women would be more exposed than men to occupational activities involving close contact with users/patients, such as in schools or hospitals [35,36,37,38,39].

Individuals between the ages of 30 and 49 were shown to be the most susceptible to reinfection, whereas older age groups were found to be more protected, with a gradual risk decline since the age of 60. In this regard, it is observed that in the Abruzzo population, people below 60’s are at a greater risk than those over sixty (adj-HR_30–59_: 2.14, 95% CI: 1.61–2.86; adj-HR_0–29_: 2.00, 95% CI: 1.53–2.60, with 60+ subjects as the reference group) [25]. The different age stratifications made it difficult to compare the younger age groups. Similarly, Jang et al. [40] demonstrated that in South Korea, taking as reference subjects between 40 and 49 years, the most at-risk groups are those 18–29 years old (OR: 1.38, 95% CI: 1.34–1.42, *p* < 0.001) and 30–39 years old (OR: 1.19, 95% CI: 1.16–1.23, *p* < 0.001); at the same time, those in their fifties and sixties have a statistically significantly lower risk of contracting a second infection (OR_50–59_: 0.84, 95% CI: 0.80–0.87; OR_60–74_: 0.86, 95% CI: 0. 83–0.89). It can be expected in older subjects to adopt better individual protective measures, such as an increase in social distance, an increase in the usage of masks, and other precautions [41].

Similarly, the results pertaining to hospitalization during the primary infection suggest that the lower risk of reinfection in previously hospitalized people is likely linked to a change in the habits and lifestyles of those with a previous severe clinical outcome.

The wide-ranging observation period allowed us to assess the effects of the spread of SARS-CoV-2 variants on reinfection frequency. This study strongly correlates reinfections with the Omicron variant: Of the 39,301 reinfection events, 36,902 (93.9%) are attributable with almost absolute certainty to the Omicron subvariants, while 1990 (5.06%) are uncertainly attributable as they occurred during the period of co-dominance between Delta and Omicron. According to our knowledge, this is one of the first studies to analyze this aspect of reinfection using such comprehensive data. In fact, some researchers have revealed that the Omicron strain may contribute much more to the rise in reinfection rates. Özüdoğru et al. [42], for instance, observed that the Omicron variant accounted for a reinfection frequency around 30 times higher than the Alpha variant and 10 folds higher than the Delta strain. In addition, our Cox multivariable regression model revealed that the probability of reinfection was at least 200% higher during the Delta, Delta-Omicron, and Omicron periods (HR > 3.40 for all times indicated, *p* < 0.001). Using the first pandemic wave as a reference, a study conducted in South Africa revealed a similar hazard risk of 1.75 (95% CI: 1.48–2.1) [43].

Despite the numerous significant results, the present study could suffer from some limitations. First, to prevent the occurrence of misclassification bias, information regarding the clinical status of participants who tested positive for SARS-CoV-2 was excluded. Unfortunately, the assessment of each record referring to the severity of the symptoms was managed by broad groups of healthcare professionals possessing varying levels of clinical expertise, and, thus, the data might have been misclassified. Regrettably, our decision to exclude data inherent to the clinical status of each participant prevented us from confirming what other studies have reported, namely that patients who experience asymptomatic or mild symptoms during their first COVID-19 infection have a higher likelihood of reinfection than those who present with a symptomatic disease [33,44,45]. Second, there was no information regarding the health status of any individual. This prevented us from assessing comorbidities, immunodeficiency disorders, and immunosuppressive therapies. It was not possible, for instance, to determine whether and how glucocorticoid treatment, which is beneficial against some of the most severe stages of COVID-19 [46], interfered with the development of a robust immune response over time. In addition, we were unable to evaluate lifestyle risk variables, including lack of use of personal protective equipment, promiscuity, and smoking and alcohol consumption. Without comorbidity data, it was impossible to correlate reinfection rates to the immunocompetence or other diseases of each participant. A further possible limitation of this research lies in the current flaws of surveillance systems. It is, in fact, useful to consider that the surveillance methods employed throughout the COVID-19 pandemic were based on the information mainly provided by symptomatic subjects or their close contacts to the competent authorities. The likely underreporting of infections and reinfections, especially at the beginning of the pandemics (wild-type and wild-type–Alpha periods), could be responsible for the sample size heterogeneity of this study. Undoubtedly, this method could lead to under-notification, especially in asymptomatic cases who were unable to report their condition due to the absence of symptoms or clinical signs of SARS-CoV-2 infection. It is crucial to highlight that a certain number of cases, particularly those who were asymptomatic, are under-reported in both the cohort of individuals who have had only one SARS-CoV-2 infection and among those who have experienced a reinfection. Consequently, considering the characteristics of the study and the fact that it is a population study, the proportions of reinfections should be considered roughly reliable and consistent.

Moreover, given the design of this study, it was not possible to perform a genomic evaluation of each nasopharyngeal swab sample; hence, the attribution of each case to a specific variant has been epidemiological-based rather than molecular-based. Since cases of reinfection confirmed with genome sequencing have been documented at an interval period ranging from 19 days [22] to 142 days [15], this absence led us to adhere to the definition postulated by the CDC and, consequently, to use a cut-off of 90 days, which may have underestimated the actual number of reinfections.

## 5. Conclusions

The findings of this study offer a sound basis to comprehend the reinfection phenomenon in Sicily, leading to the analysis of some important determinants of reinfection and emphasizing the most urgent issues that should be addressed through specific policies.

In detail, it emerged that there is a significant decrease in the risk of reinfection following vaccination, which has been and remains one of the most important tools to face SARS-CoV-2 infections. Those who received two or three mRNA vaccine doses are decisively better protected than unvaccinated subjects and those who received just one vaccine shot.

Moreover, we found that female subjects are more susceptible to SARS-CoV-2 reinfection; age groups with the highest risk of reinfection were 30–39 years and 40–49 years, suggesting that social determinants of health play a significant role; similarly, individuals hospitalized during the primary infection were at a lower risk of reinfection, and reinfection episodes have increased significantly over time, particularly with the spread of new, highly transmissible variants, with the Omicron variant reaching its peak.

In light of this, it is essential that each relevant preventative action be carried out. These actions should include encouraging yearly COVID-19 vaccination, preferably in conjunction with seasonal influenza vaccination, and recommending the adoption of personal protective measures, especially for those population subgroups that are most at risk.

## Figures and Tables

**Table 1:** Proportion of patients with SARS-CoV-2 reinfection.

	*Months*	*Overall Registered COVID-19 Cases*		*Proportion of Reinfections*	
		*n*	% of Infections on the Entire Period	*n*	% of Infections on the Entire Period
2020					
	February	8	0.00%	0	0
	March	1583	0.17%	0	0
	April	925	0.10%	0	0
	May	176	0.02%	0	0
	June	58	0.01%	0	0
	July	185	0.02%	0	0
	August	816	0.09%	0	0
	September	2417	0.27%	0	0
	October	14,160	1.56%	0	0
	November	36,648	4.05%	0	0
	December	23,890	2.64%	0	0
	*Subtotal*	*80,866*			
2021					
	January	34,206	3.78%	3	0.01%
	February	12,107	1.34%	2	0.01%
	March	18,223	2.01%	2	0.01%
	April	26,072	2.88%	8	0.02%
	May	11,543	1.27%	0	0.00%
	June	3788	0.42%	4	0.01%
	July	8813	0.97%	52	0.13%
	August	25,753	2.84%	117	0.30%
	September	14,313	1.58%	84	0.21%
	October	6826	0.75%	47	0.12%
	November	11,025	1.22%	53	0.13%
	December	51,515	5.69%	778	1.98%
	*Subtotal*	*224,184*		*1150*	
2022					
	January	193,882	21.40%	4532	11.53%
	February	106,833	11.79%	3480	8.85%
	March	139,244	15.37%	4367	11.11%
	April	99,188	10.95%	3248	8.26%
	May	57,350	6.33%	2287	5.82%
	June	4282 *	0.47% *	5028	12.79%
	July	*	*	12,798	32.56%
	August	*	*	2411	6.13%
	*Subtotal*	*600,779*		*38,151*	
	**TOTAL**	905,829	100,00%	39,301	4,34%

* The data regarding each first SARS-CoV-2 infection that occurred after 11 June 2022 are not included in the analysis. This is because the inclusion criteria specified a minimum follow-up period of 90 days from the date of the primary infection.

**Table 2:** Demographic and clinical characteristics of the Sicilian cohort by reinfection incidence.

		*Reinfection Incidence*		
	Total	No	Yes	*p*-Value
	*n* = 905,829 (% by Column)	*n* = 866,528 (% by Row)	*n* = 39,301 (% by Row)	
**Sex**				
Female	500,154 (55.2%)	476,766 (95.3%)	23,388 (4.7%)	<0.001
Male	405,675 (44.8%)	389,762 (96.1%)	15,913 (3.9%)	
**Age**				
18–29	175,388 (19.4%)	167,117 (95.3%)	8271 (4.7%)	<0.001
30–39	166,005 (18.3%)	157,028 (94.6%)	8977 (5.4%)	
40–49	176,819 (19.5%)	168,207 (95.1%)	8612 (4.9%)	
50–59	159,127 (17.6%)	152,294 (95.7%)	6833 (4.3%)	
60–69	106,029 (11.7%)	102,295 (96.5%)	3734 (3.5%)	
70–79	67,567 (7.5%)	65,885 (97.5%)	1682 (2.5%)	
80+	54,894 (6.1%)	53,703 (97.8%)	1191 (2.2%)	
**Hospitalization**				
No	879,253 (97.1%)	841,414 (95.7%)	37,839 (4.3%)	<0.001
Yes	26,576 (2.9%)	25,115 (94.5%)	1461 (5.5%)	
**Vaccination status**				
Unvaccinated	169,625 (18.7%)	157,852 (93.1%)	11,773 (6.9%)	<0.001
Incomplete vaccination	77,098 (8.5%)	67,581 (87.7%)	9517 (12.3%)	
Full vaccination cycle	354,078 (39.1%)	340,943 (96.2%)	13,135 (3.8%)	
Boosted	305,028 (33.7%)	300,152 (98.4%)	4876 (1.6%)	
**Variant dominance**				
Wild type	102,924 (11.4%)	102,921 (100%)	3 (0%)	<0.001
Wild-type–Alpha	85,077 (9.4%)	85,056 (100%)	21 (0%)	
Delta	72,238 (8%)	71,853 (99.5%)	385 (0.5%)	
Delta–Omicron	106,443 (11.8%)	104,453 (98.1%)	1990 (1.9%)	
Omicron	539,147 (59.5%)	502,245 (93.1%)	36,902 (6.9%)	

## Data Availability

The dataset generated during and/or analyzed during the current study is available from the corresponding author on reasonable request.

## References

[1] Hu T., Liu Y., Zhao M., Zhuang Q., Xu L., He Q. (2020). A Comparison of COVID-19, SARS and MERS. PeerJ.

[2] Zhu N., Zhang D., Wang W., Li X., Yang B., Song J., Zhao X., Huang B., Shi W., Lu R. (2020). A Novel Coronavirus from Patients with Pneumonia in China, 2019. N. Engl. J. Med..

[3] WHO Coronavirus (COVID-19) Dashboard. https://covid19.who.int.

[4] Graña C., Ghosn L., Evrenoglou T., Jarde A., Minozzi S., Bergman H., Buckley B.S., Probyn K., Villanueva G., Henschke N. (2022). Efficacy and Safety of COVID-19 Vaccines. Cochrane Database Syst. Rev..

[5] Amodio E., Genovese D., Mazzeo L., Martino L., Restivo V., Vella G., Calamusa G., Vitale F. (2022). Effectiveness of mRNA COVID-19 Vaccines in Adolescents Over 6 Months. Pediatrics.

[6] Prunas O., Weinberger D.M., Pitzer V.E., Gazit S., Patalon T. (2022). Waning Effectiveness of the BNT162b2 Vaccine Against Infection in Adolescents. medRxiv.

[7] Tartof S.Y., Slezak J.M., Fischer H., Hong V., Ackerson B.K., Ranasinghe O.N., Frankland T.B., Ogun O.A., Zamparo J.M., Gray S. (2021). Effectiveness of mRNA BNT162b2 COVID-19 Vaccine up to 6 Months in a Large Integrated Health System in the USA: A Retrospective Cohort Study. Lancet.

[8] Andrews N., Tessier E., Stowe J., Gower C., Kirsebom F., Simmons R., Gallagher E., Thelwall S., Groves N., Dabrera G. (2022). Duration of Protection against Mild and Severe Disease by COVID-19 Vaccines. N. Engl. J. Med..

[9] Olson S.M., Newhams M.M., Halasa N.B., Price A.M., Boom J.A., Sahni L.C., Pannaraj P.S., Irby K., Walker T.C., Schwartz S.P. (2022). Effectiveness of BNT162b2 Vaccine against Critical Covid-19 in Adolescents. N. Engl. J. Med..

[10] Tenforde M.W., Self W.H., Naioti E.A., Ginde A.A., Douin D.J., Olson S.M., Talbot H.K., Casey J.D., Mohr N.M., Zepeski A. (2021). Sustained Effectiveness of Pfizer-BioNTech and Moderna Vaccines Against COVID-19 Associated Hospitalizations Among Adults—United States, March-July 2021. Morb. Mortal. Wkly. Rep..

[11] Amicone M., Borges V., Alves M.J., Isidro J., Zé-Zé L., Duarte S., Vieira L., Guiomar R., Gomes J.P., Gordo I. (2022). Mutation Rate of SARS-CoV-2 and Emergence of Mutators during Experimental Evolution. Evol. Med. Public Health.

[12] Wang Z., Schmidt F., Weisblum Y., Muecksch F., Barnes C.O., Finkin S., Schaefer-Babajew D., Cipolla M., Gaebler C., Lieberman J.A. (2021). mRNA Vaccine-Elicited Antibodies to SARS-CoV-2 and Circulating Variants. Nature.

[13] Gaudreault N.N., Carossino M., Morozov I., Trujillo J.D., Meekins D.A., Madden D.W., Cool K., Artiaga B.L., McDowell C., Bold D. (2021). Experimental Re-Infected Cats Do Not Transmit SARS-CoV-2. Emerg. Microbes Infect..

[14] Brustolin M., Rodon J., Rodríguez de la Concepción M.L., Ávila-Nieto C., Cantero G., Pérez M., Te N., Noguera-Julián M., Guallar V., Valencia A. (2021). Protection against Reinfection with D614- or G614-SARS-CoV-2 Isolates in Golden Syrian Hamster. Emerg. Microbes Infect..

[15] To K.K.-W., Hung I.F.-N., Ip J.D., Chu A.W.-H., Chan W.-M., Tam A.R., Fong C.H.-Y., Yuan S., Tsoi H.-W., Ng A.C.-K. (2021). Coronavirus Disease 2019 (COVID-19) Re-Infection by a Phylogenetically Distinct Severe Acute Respiratory Syndrome Coronavirus 2 Strain Confirmed by Whole Genome Sequencing. Clin. Infect. Dis. Off. Publ. Infect. Dis. Soc. Am..

[16] Prado-Vivar B., Becerra-Wong M., Guadalupe J.J., Márquez S., Gutierrez B., Rojas-Silva P., Grunauer M., Trueba G., Barragán V., Cárdenas P. (2021). A Case of SARS-CoV-2 Reinfection in Ecuador. Lancet Infect. Dis..

[17] Prado-Vivar B., Becerra-Wong M., Guadalupe J.J., Marquez S., Gutierrez B., Rojas-Silva P., Grunauer M., Trueba G., Barragan V., Cardenas P. (2020). COVID-19 Re-Infection by a Phylogenetically Distinct SARS-CoV-2 Variant, First Confirmed Event in South America. https://papers.ssrn.com/sol3/papers.cfm?abstract_id=3686174.

[18] Borgogna C., de Andrea M., Griffante G., Lai A., Bergna A., Galli M., Zehender G., Castello L., Ravanini P., Cattrini C. (2021). SARS-CoV-2 Reinfection in a Cancer Patient with a Defective Neutralizing Humoral Response. J. Med. Virol..

[19] Gupta V., Bhoyar R.C., Jain A., Srivastava S., Upadhayay R., Imran M., Jolly B., Divakar M.K., Sharma D., Sehgal P. (2021). Asymptomatic Reinfection in 2 Healthcare Workers from India with Genetically Distinct Severe Acute Respiratory Syndrome Coronavirus 2. Clin. Infect. Dis..

[20] Larson D., Brodniak S.L., Voegtly L.J., Cer R.Z., Glang L.A., Malagon F.J., Long K.A., Potocki R., Smith D.R., Lanteri C. (2021). A Case of Early Reinfection With Severe Acute Respiratory Syndrome Coronavirus 2 (SARS-CoV-2). Clin. Infect. Dis..

[21] Yahav D., Yelin D., Eckerle I., Eberhardt C.S., Wang J., Cao B., Kaiser L. (2021). Definitions for Coronavirus Disease 2019 Reinfection, Relapse and PCR Re-Positivity. Clin. Microbiol. Infect. Off. Publ. Eur. Soc. Clin. Microbiol. Infect. Dis..

[22] Shastri J., Parikh S., Agrawal S., Chatterjee N., Pathak M., Chaudhary S., Sharma C., Kanakan A., Vivekanand A., Srinivasa Vasudevan J. (2021). Clinical, Serological, Whole Genome Sequence Analyses to Confirm SARS-CoV-2 Reinfection in Patients from Mumbai, India. Front. Med..

[23] Flacco M.E., Acuti Martellucci C., Baccolini V., de Vito C., Renzi E., Villari P., Manzoli L. (2022). Risk of Reinfection and Disease after SARS-CoV-2 Primary Infection: Meta-Analysis. Eur. J. Clin. Investig..

[24] Pecoraro V., Pirotti T., Trenti T. (2022). Evidence of SARS-CoV-2 Reinfection: Analysis of 35,000 Subjects and Overview of Systematic Reviews. Clin. Exp. Med..

[25] Flacco M.E., Soldato G., Acuti Martellucci C., di Martino G., Carota R., Caponetti A., Manzoli L. (2022). Risk of SARS-CoV-2 Reinfection 18 Months After Primary Infection: Population-Level Observational Study. Front. Public Health.

[26] Ren X., Zhou J., Guo J., Hao C., Zheng M., Zhang R., Huang Q., Yao X., Li R., Jin Y. (2022). Reinfection in Patients with COVID-19: A Systematic Review. Glob. Health Res. Policy.

[27] Centers for Disease Control and Prevention Common Investigation Protocol for Investigating Suspected SARS-CoV-2 Reinfection. https://www.cdc.gov/coronavirus/2019-ncov/php/reinfection.html.

[28] European Centre for Disease Prevention and Control (ECDC) Data on SARS-CoV-2 Variants in the EU/EEA. https://www.ecdc.europa.eu/en/publications-data/data-virus-variants-covid-19-eueea.

[29] Amodio E., Genovese D., Fallucca A., Ferro P., Sparacia B., D’Azzo L., Fertitta A., Maida C.M., Vitale F. (2023). Clinical Severity in Different Waves of SARS-CoV-2 Infection in Sicily: A Model of Smith’s “Law of Declining Virulence” from Real-World Data. Viruses.

[30] Cavanaugh A.M., Spicer K.B., Thoroughman D., Glick C., Winter K. (2021). Reduced Risk of Reinfection with SARS-CoV-2 after COVID-19 Vaccination—Kentucky, May–June 2021. Morb. Mortal. Wkly. Rep..

[31] Hammerman A., Sergienko R., Friger M., Beckenstein T., Peretz A., Netzer D., Yaron S., Arbel R. (2022). Effectiveness of the BNT162b2 Vaccine after Recovery from COVID-19. N. Engl. J. Med..

[32] Cohen J.I., Burbelo P.D. (2021). Reinfection With SARS-CoV-2: Implications for Vaccines. Clin. Infect. Dis..

[33] Bechmann N., Barthel A., Schedl A., Herzig S., Varga Z., Gebhard C., Mayr M., Hantel C., Beuschlein F., Wolfrum C. (2022). Sexual Dimorphism in COVID-19: Potential Clinical and Public Health Implications. Lancet Diabetes Endocrinol..

[34] Piazza M.F., Amicizia D., Marchini F., Astengo M., Grammatico F., Battaglini A., Sticchi C., Paganino C., Lavieri R., Andreoli G.B. (2022). Who Is at Higher Risk of SARS-CoV-2 Reinfection? Results from a Northern Region of Italy. Vaccines.

[35] Galasso V., Pons V., Profeta P., Becher M., Brouard S., Foucault M. (2020). Gender Differences in COVID-19 Attitudes and Behavior: Panel Evidence from Eight Countries. Proc. Natl. Acad. Sci. USA.

[36] Wenham C., Smith J., Morgan R. (2020). COVID-19: The Gendered Impacts of the Outbreak. Lancet.

[37] Bertocchi G. COVID-19 Susceptibility, Women, and Work. https://cepr.org/voxeu/columns/covid-19-susceptibility-women-and-work.

[38] Ballering A.V., Oertelt-Prigione S., Olde Hartman T.C., Rosmalen J.G.M., Boezen M., Mierau J.O., Franke L.H., Dekens J., Deelen P., Lanting P. (2021). Sex and Gender-Related Differences in COVID-19 Diagnoses and SARS-CoV-2 Testing Practices During the First Wave of the Pandemic: The Dutch Lifelines COVID-19 Cohort Study. J. Womens Health.

[39] Hawkes S., Pantazis A., Purdie A., Gautam A., Kiwuwa-Muyingo S., Buse K., Tanaka S., Borkotoky K., Sharma S., Verma R. (2022). Sex-Disaggregated Data Matters: Tracking the Impact of COVID-19 on the Health of Women and Men. Econ. Polit..

[40] Jang E.J., Choe Y.J., Yun G.-W., Wang S., Cho U.J., Yi S., Lee S., Park Y.-J. (2022). Reinfection with SARS-CoV-2 in General Population, South Korea; Nationwide Retrospective Cohort Study. J. Med. Virol..

[41] Pilz S., Theiler-Schwetz V., Trummer C., Krause R., Ioannidis J.P.A. (2022). SARS-CoV-2 Reinfections: Overview of Efficacy and Duration of Natural and Hybrid Immunity. Environ. Res..

[42] Özüdoğru O., Bahçe Y.G., Acer Ö. (2023). SARS CoV-2 Reinfection Rate Is Higher in the Omicron Variant than in the Alpha and Delta Variants. Ir. J. Med. Sci..

[43] Pulliam J.R.C., van Schalkwyk C., Govender N., von Gottberg A., Cohen C., Groome M.J., Dushoff J., Mlisana K., Moultrie H. (2022). Increased Risk of SARS-CoV-2 Reinfection Associated with Emergence of Omicron in South Africa. Science.

[44] Iwasaki A. (2021). What Reinfections Mean for COVID-19. Lancet Infect. Dis..

[45] Van Elslande J., Vermeersch P., Vandervoort K., Wawina-Bokalanga T., Vanmechelen B., Wollants E., Laenen L., André E., van Ranst M., Lagrou K. (2021). Symptomatic Severe Acute Respiratory Syndrome Coronavirus 2 (SARS-CoV-2) Reinfection by a Phylogenetically Distinct Strain. Clin. Infect. Dis. Off. Publ. Infect. Dis. Soc. Am..

[46] Salton F., Confalonieri P., Campisciano G., Cifaldi R., Rizzardi C., Generali D., Pozzan R., Tavano S., Bozzi C., Lapadula G. (2022). Cytokine Profiles as Potential Prognostic and Therapeutic Markers in SARS-CoV-2-Induced ARDS. J. Clin. Med..

